# Effects of nano silicon fertilizer on the lodging resistance characteristics of wheat basal second stem node

**DOI:** 10.1186/s12870-024-04735-z

**Published:** 2024-01-18

**Authors:** Min Yang, Shuang Chen, Kui Chao, Cexun Ji, Yan Shi

**Affiliations:** Dryland-technology Key Laboratory of Shandong Province, College of Agronomy, Qingdao Agricultural University, Qingdao, 266109 China

**Keywords:** *Triticum aestivum L.*, Nano silicon, Lodging resistance, Lignin accumulation, Gene expression

## Abstract

The application of nano fertilizers is one of the hotspots in current agricultural production. In this study, nano silicon materials were mixed with compound fertilizers to make nano silicon fertilizer. The effects of different amounts of nano silicon application on the breaking-resistance strength, lodging-resistance index, lignin accumulation, lignin synthesis related enzymes, and the relative expression of lignin synthesis related genes in the second stem node of wheat were mainly studied. Four treatments were set up: CK (750 kg·ha^−1^ compound fertilizer), T1 (750 kg·ha^−1^ compound fertilizer + 0.9 kg·ha^−1^ nano silicon), T2 (750 kg·ha^−1^ compound fertilizer + 1.8 kg·ha^−1^ nano silicon), T3 (750 kg·ha^−1^ compound fertilizer + 2.7 kg·ha^−1^ nano silicon). The results of the two-year experiment showed that the breaking-resistance strength, lodging-resistance index, lignin accumulation in the second stem node of wheat treated with nano silicon fertilizer were higher than CK. In the first year of the experiment, the lignin accumulation of T2 was 130.73%, 5.14% and 7.25% higher than that of CK, T1 and T3 respectively at the maturity stage. In the second year of the experiment, the lignin accumulation of T2 was 20.33%, 11.19% and 9.89% higher than that of CK, T1 and T3 respectively at the maturity stage. And the activities of PAL, 4CL, CAD, and related gene expression levels were also higher than CK. And among them, T2 performed the best, indicating that the application of nano silicon fertilizer is beneficial for improving the lodging resistance of wheat stems and is of great significance for improving the quality of wheat.

## Introduction

Wheat (*Triticum aestivum L.*) is one of the most important food crops in the world. In 2022, the yield of wheat in China reached 1377.3 × 10^8^ kg [1]. As an important source of nutrients for crop growth, chemical fertilizer contributes more than 50% to the grain yield increase in China [2], which plays an important role in ensuring high yield and high quality of grain crops. However, the utilization rate of fertilizers in cereal crops in China is generally low [3], and excessive application of fertilizers will also lead to increased production costs, environmental pollution and other problems, so how to efficiently apply fertilizers to promote high yield and high quality of crops is one of the main problems facing agricultural development [4].

Nanotechnology is a new technology that emerged in the late 1980s. At present, nanotechnology, biotechnology and Information science technology are called the three pillar industries of social and economic development in the twenty-first century [5]. At present, nano-fertilizer is developing rapidly and has broad prospects [6], and its application in agriculture is becoming more and more extensive. Nanomaterials have excellent adsorption and colloidal stability as coating materials. Adding nanomaterials to fertilizers can effectively control the release of fertilizer nutrients. At the same time, nanoparticles can improve the absorption capacity of crop roots, which is more conducive to the absorption and utilization of nutrients by crops [7]. Silicon fertilizer was listed as the fourth largest element fertilizer after nitrogen, phosphorus and potassium by the international soil community [8]. Silicon is the second most abundant element on the earth's surface [9], and some studies have shown that silicon can significantly improve the stress resistance of plants, protect crops from pathogen infection, improve the ability to resist fungal diseases, and enhance the toughness of plants, which is very beneficial to the healthy growth of crops[10–12]. Silicon element is non-corrosive and sustainable, and silicon fertilizer is considered to be environmentally friendly and sustainable agricultural fertilizer [13], which has been paid more and more attention in agricultural practice. Compared with traditional fertilizer, nano-fertilizer has many advantages, including variable solubility, enhanced effective concentration, time-controlled emission, and less ecotoxicity [14].

Lodging is a limiting factor for wheat high yield. In Huang-Huai-Hai wheat area, about 10% of the area is lodging every year, and more than 20% in severe years, and even no harvest [15]. Breeding practice shows that stem strength is a very important trait to determine the lodging resistance of varieties [16], and there is a significant positive correlation between stem strength and lignin content [17]. Lignin plays an important role in cell wall structural integrity, stem strength, transport, mechanical support and plant pathogen defense [18]. The content of lignin is closely related to the lodging resistance of stems. Meng et al. [19] found that varieties with high lignin content usually have strong lodging resistance. Related studies have shown that the lignin content of stems is related to the activities of phenylalanine ammonia lyase (PAL), cinnamyl alcohol dehydrogenase (CAD), and coumaric acid coenzyme A ligase (4CL). These three enzymes have a certain regulatory effect on the synthesis of lignin, and their activity can reflect the lodging resistance of wheat stems [20].

Some studies have found that in rice (*Oryza. sativa L.*), the application of silicon fertilizer will increase the thickness of stem wall, the size of vascular bundles and peroxidase activity, thereby improving stress resistance, stem strength and preventing lodging [18, 21]. Pei et al. [22] found that the application of nano silicon fertilizer had the effect of increasing yield and improving quality of amaranth. Sun et al. [23] found that nano silicon can be absorbed by plants, and can be transported and migrated between different tissues along with water or nutrients through xylem and phloem of plants, and accumulated in different parts of plants. In some cases, nano silicon can participate in the construction of plants' own tissues or organelles, affect the synthesis of physiologically active substances, participate in and regulate physiological metabolic activities, and thus affect the growth and development of plants.

At present, nano silicon fertilizer has been studied more on rice, flue-cured tobacco (*Nicotianatabacum*) and other crops [24, 25], but less on wheat crops. The cells of the second stem node at the base of wheat are closely arranged, with a large number of vascular bundles, thick thin-walled tissue, and high mechanical strength, which are closely related to the lodging resistance of the stem [26]. The advantages of nanomaterials in agricultural production and fertilizer release have brought new opportunities for agricultural development and provided an important way to improve the utilization efficiency of fertilizers and maintain the sustainable development of agricultural ecosystems [27–29]. Therefore, in this study, nano silicon materials were mixed with compound fertilizer to make nano silicon fertilizer, mainly to explore the effects of applying nano silicon fertilizer on the breaking-resistance strength, lodging-resistance index and lignin accumulation, as well as the enzymes activity related to lignin synthesis and relative expression of related genes of wheat basal second stem node at different stages.

## Materials and design

### Experimental materials

**Fig. 1:**
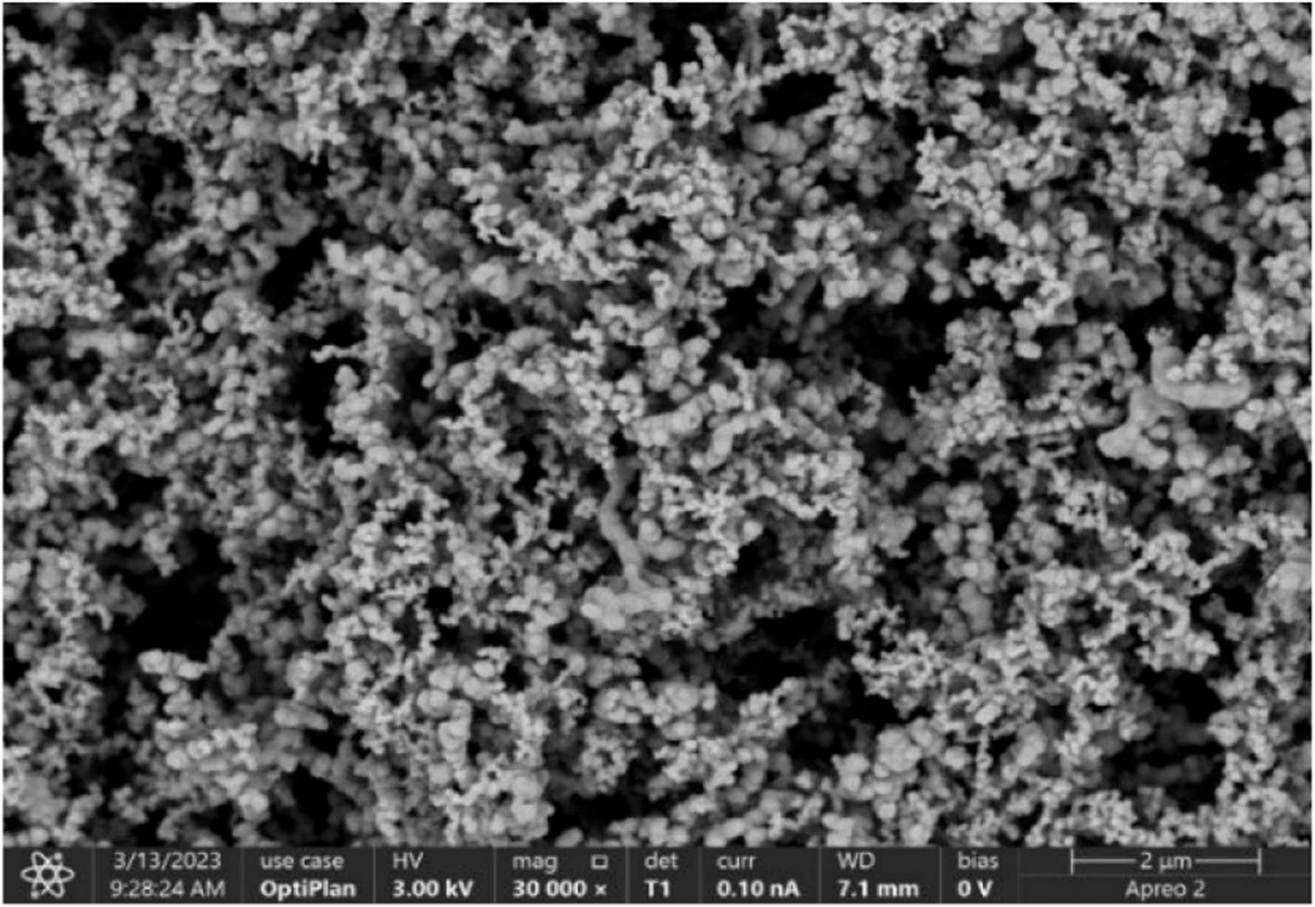
Scanning electron microscopy of nano silicon

The wheat variety Taimai 198 was used in the experiment. The compound fertilizer used was Sinochem Compound Fertilizer (total nutrients ≥ 45%; N-P_2_O_5_-K_2_O was 18:20:7), which was applied as the basal fertilization. The nano silicon was produced by Beijing Deke DaoJin Science And Technology Co., Ltd, the particle size of nano silicon is 35 nm, the specific surface area is 42.4 m^2^/g and the silicon content is 55.29%. The scanning electron microscope image of nano silicon is shown in Fig. 1. Before sowing, a certain amount of nano silicon and compound fertilizer were weighed and mixed well to form nano silicon fertilizer, which was evenly scatter on the soil surface and ploughed into the soil by tillage.

### Experimental design

The experiment was a two-year field experiment, which was conducted in Weifang Changyi Experimental Base of Qingdao Agricultural University (N37°0 ′ 27.24 ″, E119°22 ′ 15.79 ″) from October 2020 to June 2021 and October 2021 to June 2022. The soil type of the field experiment was sandy loam soil. The basic soil productivity before sowing is shown in Table 1. The determination method referred to *Soil and Agricultural Chemistry Analysis *[30], the content of alkali hydrolyzable nitrogen in soil was determined by alkali solution diffusion method, the content of available phosphorus was determined by molybdenum antimony colorimetric method, the content of available potassium was determined by ammonium acetate extraction flame spectrophotometry, the content of available silicon was determined by silicon molybdenum blue colorimetric method [31], the content of organic matter was determined by hydration heat potassium dichromate oxidation method, and the pH was determined by pH meter. The experiment was conducted using a randomized complete block design (RCBD), the size of each plot was 3 × 5 = 15 m^2^, and each treatment was repeated 3 times, and the sowing method was mechanical drilling. The experiment set up 4 treatments, and the specific experimental design is shown in Table 2. The first-year experiment was sown on October 16, 2020, with a seeding rate of 187.5 kg·ha^−1^, and harvested on June 14, 2021. The second-year experiment was sown on October 11, 2021, with a seeding rate of 187.5 kg·ha^−1^, and harvested on June 12, 2022. Planted corn in the field plot from the wheat harvest in the first year to the reseeding. The climatic data during the field experiment shown in Table 3. During the whole growth stage, the wheat crop did not receive irrigation and fertilizer topdressing.

**Table 1:** Basic soil productivity

Year	Alkaline hydrolysis nitrogen (mg·kg^−1^)	Available phosphorus (mg·kg^−1^)	Available potassium (mg·kg^−1^)	Available silicon (mg·kg^−1^)	Organic matter (g·kg^−1^)	pH
2020	65.97	20.56	250.37	88.27	13.30	8.08
2021	70.28	19.58	243.87	90.28	15.38	7.94

**Table 2:** The treatments of field experiment

Treatment	Treatment Content	Content of Each Component	
		**Compound fertilizer (kg·ha**^**−1**^**)**	**Nano silicon (kg·ha**^**−1**^**)**
CK	Compound fertilizer	750	0
T1	Compound fertilizer + Nano silicon	750	0.9
T2	Compound fertilizer + Nano silicon	750	1.8
T3	Compound fertilizer + Nano silicon	750	2.7

**Table 3:** Weather conditions during the field experiment

Month	2020–2021		2021–2022		
	Average temperature (℃)	Total precipitation (mm)	Average temperature (℃)		Total precipitation (mm)
**Oct**	15.7	18.4	14.9	114.3	
**Nov**	9.1	14.7	8.9	45.5	
**Dec**	0.3	11.9	2.6	2.0	
**Jan**	-0.4	1.4	-0.7	1.6	
**Feb**	1.2	4.0	0.8	0.6	
**Mar**	9.4	9.9	7.9	32.7	
**Apr**	13.8	32.4	14.8	20.7	
**May**	22	5.3	20.3	11.5	
**Jun**	26.5	32.3	23.6	68.5	

## Measurement items and methods

### Silicon content in aboveground parts of wheat

Samples were taken at the anthesis stage, filling stage and maturity stage of wheat. The aboveground parts of 10 wheat tillers with group representation and consistent growth were cut from each plot. The wheat plant samples were placed in an oven at 105 °C for 30 min, dried to constant weight at 75℃, and then ground into powder with a grinding prototype, weighed a certain weight sample, added hydrochloric acid, nitric acid and hydrofluoric acid in proportion, heated for about 1 h in a constant temperature graphite digestion furnace at 125 °C, constant volume, and take the supernatant for measurement using a plasma emission spectrometer (ICP).

### lodging resistance characteristics of wheat basal second stem node

The sampling time and method were the same as 3.1. After the wheat samples were brought back to the laboratory, the breaking-resistance, lodging-resistance index and lignin accumulation were measured immediately. The breaking-resistance strength of wheat stems was measured using a stem strength tester (YYD-1a, Hangzhou Top Instrument Co., Ltd., Hangzhou, China) (unit N). The height of the center of gravity is the distance from the base to the equilibrium point of wheat (unit cm). Lodging-resistance index was calculated according to the formula: lodging-resistance index = breaking-resistance strength / center of gravity height [32]. The determination of lignin accumulation was based on the method of Zheng et al. [33]. Wheat stalks were placed in a mortar and grinded into powder with liquid nitrogen. Then used ethanol to extract overnight, and then used ethanol and n-hexane mixed solution to extract, dissolved with ethyl bromide glacial acetic acid, and then dissolved with hydroxylamine hydrochloride and glacial acetic acid, and finally determined the absorbance at 280 nm.

### Enzyme activity related to lignin synthesis

#### PAL activity

The sampling time was the same as 3.1. The second stem node of 3 wheat plants with uniform growth were randomly cut from each plot, wrapped in tin paper, quickly placed in liquid nitrogen, brought back to the laboratory, and placed in a refrigerator at − 80 °C for later use. The determination of PAL activity was referred to *Experimental Guide for Plant Physiology (5th edition)* [34]. 0.5 g wheat stem was weighed and placed in a mortar, and 6 mL sodium borate-boric acid buffer (0.05 mol·L^−1^, pH 8.8, containing 5 mmol·L^−1^ mercaptoethanol, 1 mmol·L^−1^ EDTA, and a little PVP) was added. The mixture was ground to homogenate under ice bath, transferred into a 10 mL centrifuge tube, and then centrifuged at 10,000 r for 15 min at 4 °C. The supernatant was the enzyme extract. 0.2 mL of the extract was added to 1 mL of L-phenylalanine (0.1 mol·L^−1^) prepared by 0.1 mol·L^−1^ sodium borate buffer and 2.8 mL of distilled water, shaken well, reacted in a water bath at 37 °C for 30 min, and immediately put into boiling water to stop the reaction; the absorbance was measured at 290 nm, with 1 mL of distilled water instead of the substrate for the same reaction as a control. The enzyme specific activity (U mg^−1^ FW) was calculated based on the change of A290 value per hour of 0.01 as a unit of enzyme activity.

#### 4CL activity

The activity of 4CL was determined according to Knobloch 's method [35]. 5 g wheat stem was weighed and placed in a glass mortar in an ice bath. 10 mL Tris–HCl buffer (0.05 mol·L^−1^, pH 8.0, containing 0.014 mol·L^−1^ mercaptoethanol and 30% glycerol) was added, and an appropriate amount of quartz sand was added. The mixture was ground to homogenate, transferred to a 10 mL centrifuge tube, centrifuged at 10,000 r at 4 °C for 15 min, and the supernatant was the enzyme extract. 0.4 mL of the extract was added to 3 mL of the reaction solution (5 μmol·mL^−1^ coumaric acid, 50 μmol·mL^−1^ ATP, 1 μmol·mL^−1^ CoA-SH, 15 μmol·mL^−1^ MgSO_4_·7H_2_O). The mixture was placed in a constant temperature water bath at 40 °C for 10 min, and the absorbance was measured at 333 nm. The same reaction was performed with 1 mL of distilled water instead of the substrate as a control. The enzyme specific activity (U mg^−1^ FW) was calculated based on the change of A333 value per hour of 0.01 as a unit of enzyme activity.

#### CAD activity

The activity of CAD was determined by the method of Morrison et al. [36]. 0.4 g wheat stem was weighed, and 3 mL buffer (0.1 mmol·mL^−1^ phosphate buffer, containing 15 mmol·mL^−1^ mercaptoethanol, 1 mmol·mL^−1^ EDTA, pH 6.25) was added. The mixture was ground under ice bath to homogenize, centrifuged at 10,000 r at 4 °C for 20 min, and the supernatant was the enzyme extract. The 0.5 mol·mL^−1^ phosphate buffer, 2 mol·mL^−1^ NADP, 1 mol·mL^−1^ trans-cinnamic acid and enzyme extract were mixed well, and reacted in a constant temperature water bath at 37 °C for 30 min. Then the absorbance at 340 nm was measured, and the enzyme extract after boiling for 1 min was used as the control. The enzyme specific activity (U mg^−1^ FW) was calculated based on the change of A340 value per hour of 0.01 as a unit of enzyme activity.

### Genes relative expression related to lignin synthesis

The wheat basal second stem node was used as the experimental material. The kits used to determine the relative expression of genes related to lignin synthesis were purchased from Ecoray Biological Company, including SteadyPure Plant RNA Extraction Kit, EVO M-MLV RT Mix Kit Reverse Transcription Kit and Green Pro Taq Hs Premixed qPCR Kit. The primer sequence of gene referred to the primer sequence of Dong He et al. [15], as shown in Table 4. The test results were processed by 2^−△△Ct^ method [37].

**Table 4:** Gene primer sequence

**Gene**	5'-3'	
*ACTIN*	F	GGGACCTCACGGATAATCTAATG
	R	CGTAAGCGAGCTTCTCCTTTAT
*TaPAL*	F	CATCTTGGAGGGAAGCTCATAC
	R	GACTTGGTGGCAAATCGAATAAC
*Ta4CL*	F	TGCACACTGGAGACATTGGC
	R	TTCGAGTTCCGCAGGAGGTA
*TaCAD*	F	GAGGTCGTCAAGATGGAC
	R	CTAGCTCTTTCTCCCTCTG

### Data processing

The experimental data were collected using Excel 2019. The variance analysis and difference significance test (*P* < 0.05) were analyzed using SPSS 24 software [38], Duncan's new multiple range method was used for multiple comparisons. The figures were drawn using Origin 2021 software.

## Results

### Effect of nano silicon fertilizer on silicon content in aboveground parts of wheat

**Fig. 2:**
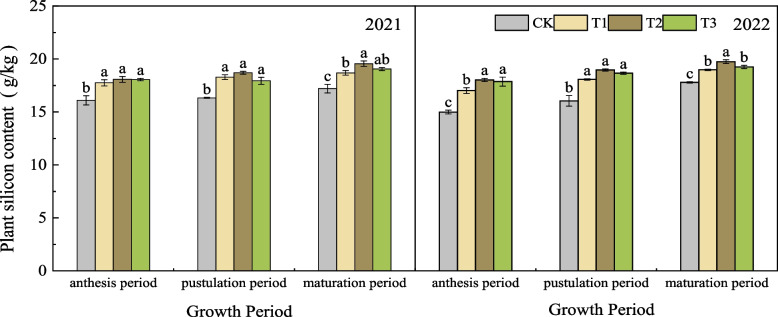
Effects of nano silicon fertilizer on plant silicon content of wheat

As shown in Fig. 2, in the two-year experiment. At each stage, the silicon content in the treatments treated with nano silicon fertilizer was higher than that in CK. In each stage of 2021, the silicon content of T2 was higher or significantly higher than the other three treatments. At maturity stage, the silicon content of T1, T2, and T3 was 8.64%,13.66%, and 10.73% higher than that of CK, respectively. In 2022, the silicon content in each stage showed T2 > T3 > T1 > CK. At maturity, the silicon content in T1, T2, and T3 was 6.69%, 10.98%, and 8.21% higher than that in CK, respectively.

### Effects of nano silicon fertilizer on the lodging resistance characteristics of wheat basal second stem node

#### Effects of nano silicon fertilizer on breaking-resistance strength of wheat basal second stem node

**Fig. 3:**
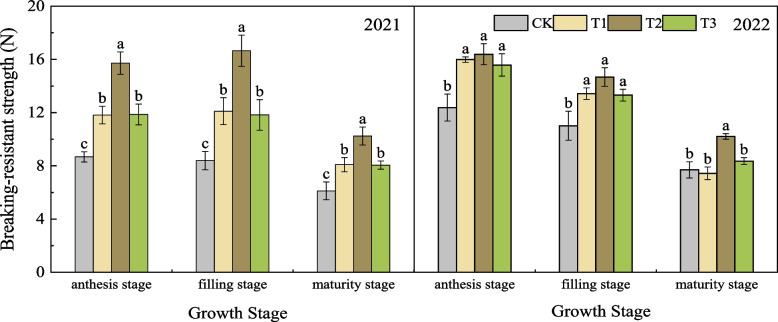
Breaking-resistant strength of wheat under different treatments

As shown in Fig. 3, in 2021, the stem breaking-resistance strength of wheat treated with nano silicon fertilizer was significantly higher than that of CK at anthesis stage, filling stage and maturity stage. Among them, T2 showed the best performance, and the breaking-resistance strength was significantly higher than that of CK, T1 and T3. From filling stage to maturity stage, the breaking-resistance strength of each treatment showed a decreasing trend, and the decreasing range was T2 > T1 > T3 > CK, which decreased by 38.48%, 33.17%, 31.88% and 27.04% respectively, but the breaking-resistance strength of T2 was still the strongest. At the maturity stage, the breaking-resistance strength of T2 was 67.38%, 26.65% and 27.18% higher than that of CK, T1 and T3, respectively. In 2022, the change trend of wheat breaking-resistance strength of each treatment was different from that of the previous year, showing a gradual downward trend. Among them, the breaking-resistance strength of T2 was still the strongest, and higher or significantly higher than that of the other three treatments. At maturity stage, the breaking-resistance strength of T2 was 32.56%, 37.19% and 22.14% higher than that of CK, T1 and T3, respectively.

#### Effects of nano silicon fertilizer on lodging-resistance index of wheat basal second stem node

**Fig. 4:**
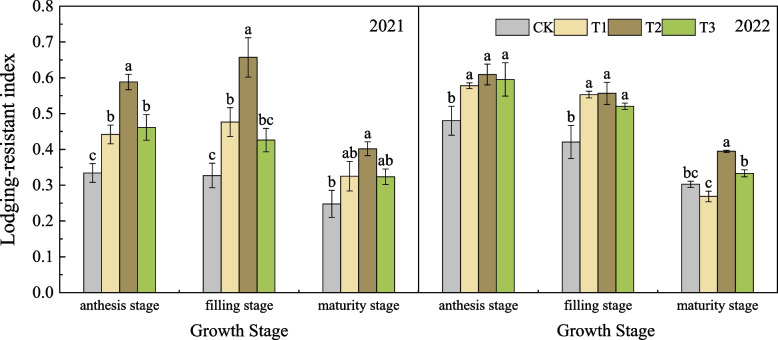
Lodging-resistant index of wheat under different treatments

As shown in Fig. 4, in 2021, the lodging-resistance index of wheat treated with nano silicon fertilizer from anthesis stage to maturity stage was higher or significantly higher than that of CK. Among them, the lodging-resistance index of T2 was the highest. At maturity stage, the lodging-resistance index of T1, T2 and T3 was 31.05%, 62.10% and 30.65% higher than that of CK, respectively. In 2022, the change trend of lodging resistance index of wheat in each treatment was different from that in the previous year, showing a decreasing trend, but the lodging-resistance index of T2 was still the highest in each stage. From filling stage to maturity stage, the lodging-resistance index of T2 decreased the least. At maturity stage, the lodging-resistance index of T2 was 32.33%, 47.04% and 19.22% higher than that of CK, T1 and T3, respectively.

#### Effects of nano silicon fertilizer on lignin accumulation of wheat basal second stem node

**Fig. 5:**
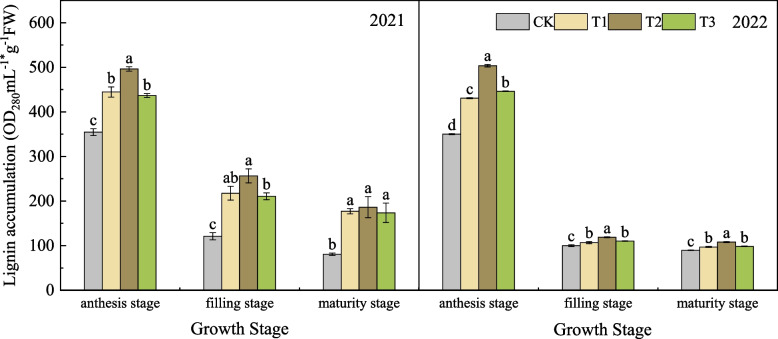
Lignin accumulation of wheat under different treatments

As shown in Fig. 5, in 2021, with the advancement of the growth stage, the lignin accumulation of wheat in each treatment showed a gradually decreasing trend. From the anthesis stage to the filling stage, the decreasing range of lignin accumulation in each treatment was CK > T3 > T1 > T2, which decreased by 65.90%, 51.77%, 51.05% and 48.31% respectively. From the filling stage to the maturity stage, the decreasing range of lignin accumulation in each treatment was CK > T2 > T1 > T3, which decreased by 33.26%, 27.39%, 18.59% and 17.54% respectively. At each stage after anthesis, the lignin accumulation of wheat treated with nano silicon fertilizer was higher or significantly higher than that of CK, and T2 was the highest. In 2022, the change trend of lignin accumulation in each treatment was the same as that in the previous year, showing a decreasing trend. The decrease from anthesis stage to filling stage was large, and the decrease from filling stage to maturity stage was small. However, the lignin accumulation of T2 in each stage was the highest and significantly higher than that of other treatments. At maturity stage, the lignin accumulation of T2 was 20.33%, 11.19% and 9.89% higher than that of CK, T1 and T3, respectively.

### Effects of nano silicon fertilizer on the enzyme activity related to lignin synthesis of wheat basal second stem node

#### Effects of nano silicon fertilizer on the PAL activity of wheat basal second stem node

**Fig. 6:**
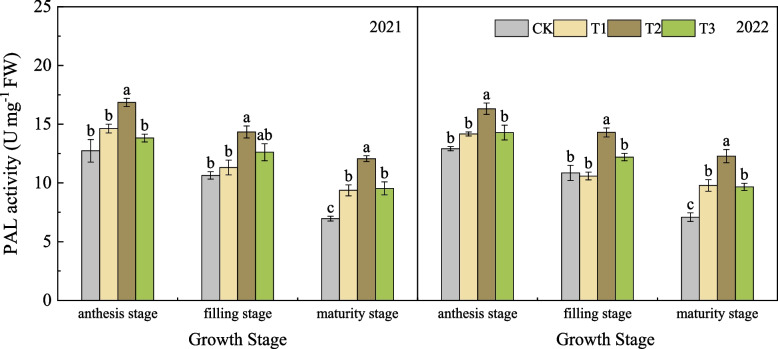
PAL activity of wheat under different treatments

As shown in Fig. 6, in 2021, the PAL activity of wheat in each treatment showed a gradually decreasing trend from anthesis stage to maturity stage. From the filling stage to the maturity stage, the decreasing range of PAL activity in each treatment was CK > T3 > T1 > T2, which decreased by 34.58%, 24.44%, 17.23% and 15.38% respectively. The decrease of PAL activity in T2 was the smallest and remained at a high level from anthesis stage to maturity stage, which was higher than the other three treatments. In 2022, except for the PAL activity of T1 was slightly lower than that of CK at the filling stage, the PAL activity of the treatments treated with nano silicon fertilizer at each stage after anthesis was higher or significantly higher than that of CK. The PAL activity of T2 was always the highest and significantly higher than that of CK, T1 and T3. At maturity stage, the PAL activity of T1, T2 and T3 was 38.14%, 73.37% and 36.35% higher than that of CK, respectively.

#### Effects of nano silicon fertilizer on the 4CL activity of wheat basal second stem node

**Fig. 7:**
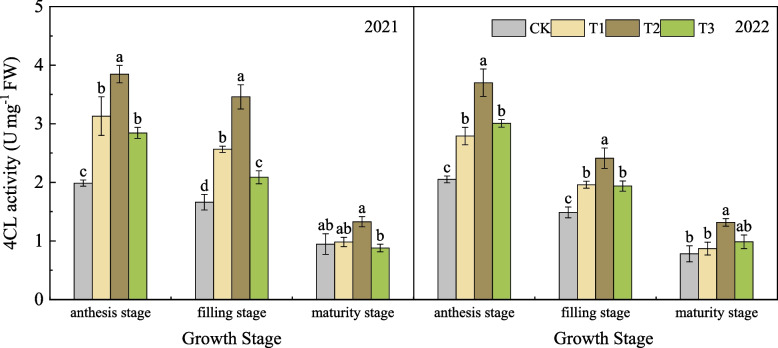
4CL activity of wheat under different treatments

As shown in Fig. 7, in 2021, the 4CL activity of wheat in each treatment showed a gradually decreasing trend from anthesis stage to maturity stage. The 4CL activity of the treatments treated with nano silicon fertilizer was higher or significantly higher than that of CK. Among them, the 4CL activity of T2 remained the highest, which was higher than that of the other three treatments. At the maturity stage, the 4CL activity of T2 was 40.13%, 34.99% and 50.80% higher than that of CK, T1 and T3, respectively. In 2022, the change trend of 4CL activity in each treatment was basically the same as that in the previous year, showing a gradually decreasing trend. The 4CL activity of the treatments with nano silicon fertilizer was higher than that of CK. From the anthesis stage to filling stage, the 4CL activity of CK, T1, T2, T3 decreased by 27.57%, 29.75%, 34.78% and 35.58%, respectively. but the 4CL activity of T2 was always at a high level. At maturity stage, the 4CL activity of T2 was 68.85%, 51.38% and 33.43% higher than that of CK, T1 and T3, respectively.

#### Effects of nano silicon fertilizer on the CAD activity of wheat basal second stem node

**Fig. 8:**
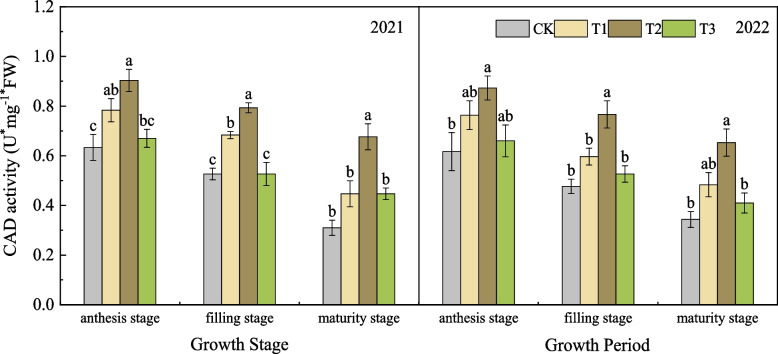
CAD activity of wheat under different treatments

As shown in Fig. 8, in the two-year experiment of 2021 and 2022, the change trend of CAD activity of wheat in each treatment was basically the same, showing a gradually decreasing trend, and the CAD activity of T1, T2 and T3 treatments with nano silicon fertilizer was higher or significantly higher than that of CK, and the CAD activity of T2 was always at a high level. At maturity stage in 2021, the CAD activity of T2 was significantly higher than that of the other three treatments, which was 118.39%, 51.45% and 51.45% higher than that of T1, T2 and T3, respectively. At maturity stage in 2022, the CAD activity of T2 was higher than that of T1 and significantly higher than that of CK and T3, which was 90.38%, 35.20% and 59.27% higher than that of T1, T2 and T3, respectively.

### Effects of nano silicon fertilizer on the relative expression of lignin synthase related genes of wheat basal second stem node

**Fig. 9:**
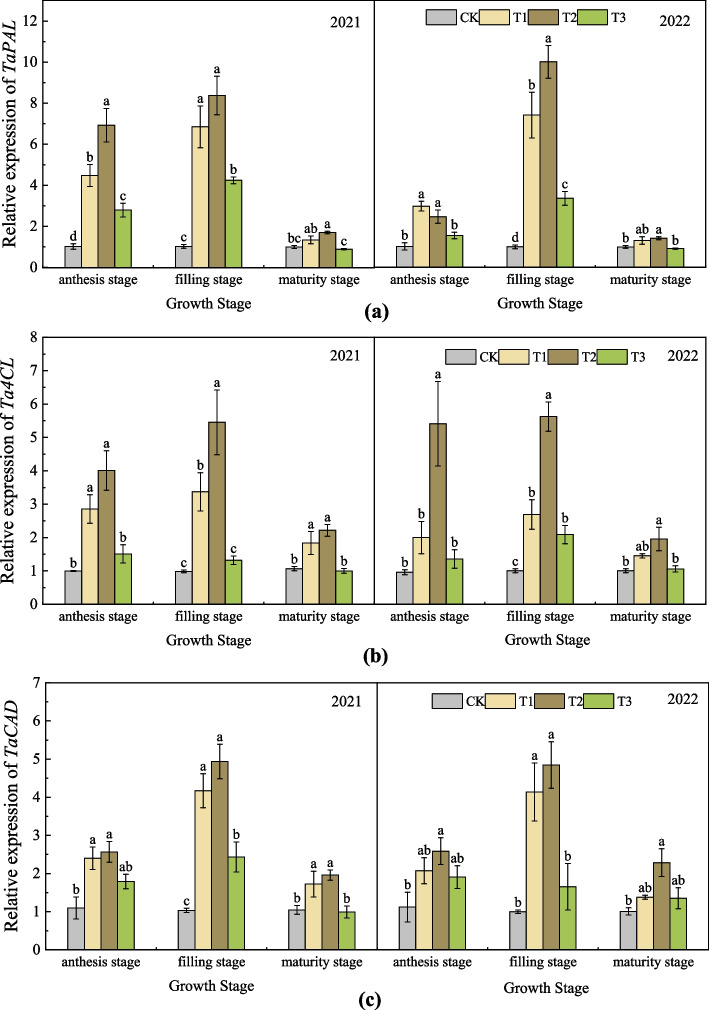
Relative expression of (**a**)TaPAL, (**b**) Ta4CL and (**c**) TaCAD of different treatments

As shown in Fig. 9 (a), in the two-year experiment of 2021 and 2022, the relative expression of *TaPAL* gene in all treatments increased first and then decreased from anthesis stage to filling stage and then to maturity stage. During the anthesis stage and filling stage, the relative expression level of *TaPAL* in the treatments treated with nano silicon fertilizer was higher or significantly higher than that in CK. At maturity stage, the relative expression level of *TaPAL* gene in each treatment was T2 > T1 > CK > T3. During the filling stage, the relative expression level of *TaPAL* is the highest. In the filling stage of 2021, the relative expression level of *TaPAL* in T2 was about 8 times that of CK. In the filling stage of 2022, the relative expression level of *TaPAL* in T2 was about 10 times that of CK. At maturity stage, the relative expression level of *TaPAL* is relatively low, but T2 was still higher than the other three treatments. As shown in Fig. 9 (b), in the two-year experiment, the relative expression level of *Ta4CL* in all treatments showed a roughly increasing and then decreasing trend at each stage after anthesis. In each stage of 2021, the relative expression level of *Ta4CL* in T2 was the highest, and the relative expression levels of *Ta4CL* in T1 and T2 was significantly higher than that in CK. The relative expression level of *Ta4CL* in T3 was not significantly different from that in CK. The changes in the relative expression level of *Ta4CL* in each treatment were basically consistent with the previous year. As shown in Fig. 9 (c), in the two-year experiment, the relative expression of *TaCAD* gene in each treatment at each stage after anthesis was basically the same as that of *TaPAL* and *Ta4CL*, showing a trend of increasing first and then decreasing. In the 2021 experiment, the relative expression of *TaCAD* in T1 and T2 was significantly higher than that of CK in each stage. In the 2022 experiment, the relative expression of *TaCAD* in each stage was T2 > T1 > T3 > CK. In the two-year experiment, the relative expression of *TaCAD* reached the lowest at maturity stage. At maturity stage in 2021, the relative expression of *TaCAD* in T2 was 87.20%, 13.56% and 97.38% higher than that of CK, T1 and T3, respectively. At maturity stage in 2022, the relative expression of *TaCAD* in T2 was 126.56%, 65.65% and 68.96% higher than that of CK, T1 and T3, respectively.

## Discussion and conclusion

Stem lodging is one of the main factors restricting the high yield of wheat, which poses a serious threat to the realization of wheat high yield. It is of great significance to study the lodging resistance of wheat stems for high and stable yield of wheat. Breaking-resistance strength is the most important factor to determine stem characteristics, and stem characteristics are the most important factor to determine whether the stem is lodging [39]. The results of this experiment showed that the stem breaking-resistance strength and lodging-resistance index of wheat treated with nano silicon fertilizer were higher than those of CK. Yang et al. [40] found that the length of the second and third stem nodes of oats after applying silicon fertilizer was shortened, the stem diameter was thickened, the wall thickness was thickened, the lodging resistance was enhanced, and the lodging area of oats was effectively reduced. The experimental results of Zhang Zhiwei et al. [41] also showed that spraying silicon fertilizer can enhance the mechanical strength of wheat basal internodes, thus enhancing the lodging resistance of wheat. These results were consistent with the results in this experiment. As one of the main components of the cell wall, lignin is filled in the cellulose framework to enhance the mechanical strength of the plant, and its content is significantly positively correlated with the breaking-resistance strength and lodging-resistance index [42]. The results of this experiment showed that with the advancement of the growth stage after anthesis, the lignin accumulation of wheat in each treatment gradually decreased, but the lignin accumulation of T1, T2 and T3 treatments with nano silicon fertilizer was significantly higher than that of CK in each stage, indicating that the application of nano silicon fertilizer could increase the wheat lignin accumulation, thereby improving the lodging resistance of wheat. Fan Yongyi et al. [43] found that the application of silicon-potassium fertilizer can increase the lignin content of rice basal second stem node, and significantly enhance the lodging resistance of rice plants.

4CL, CAD and PAL are important enzymes in the process of lignin synthesis in gramineous plants and play an important role in plant resistance response [44]. The biosynthesis of lignin monomer is obtained through the phenylpropanoid pathway. As a rate-limiting enzyme for phenylpropanoid metabolism, PAL can use L-phenylalanine as a suonbstrate to catalyze the synthesis of ammonia and trans-cinnamic acid, providing a substrate for the synthesis of secondary metabolites such as downstream lignin [45, 46]. 4CL plays a pivotal role in linking lignin precursors and various branch pathways, which helps to guide carbon flow to lignin biosynthesis or flavonoid biosynthesis [47]. CAD is the key enzyme in the last step of lignin synthesis pathway [48, 49]. Chen et al. [20] found that PAL activity was significantly positively correlated with lignin content in wheat stems, and CAD activity was significantly positively correlated with lignin content and breaking-resistant strength. The correlation coefficients were 0.85 (*P* < 0.05) and 0.72 (*P* < 0.05), respectively. Wang et al. [39] found that the 4CL activity and lignin content of highland barley stems were significantly positively correlated, and the correlation coefficient was 0.856 (*P* < 0.05). The increase of 4CL activity could enhance the lodging resistance of stems. Tang et al. [50] found that the expression abundance of *PAL* gene in xylem and phloem fiber tissues of poplar was higher, while the expression abundance in tissues with lower lignin content, such as stem, leaf and pith, was lower. Meng et al. [51] found that *Ta4CL* gene is mainly involved in the biosynthesis of lignin, and also affects the growth and development of plants and participates in the response to hormone treatment. Ma et al. [52] found that the wheat *TaCAD1* gene was highly expressed in the stem, with trace expression in the leaves and almost no expression in the roots, and compared with the non-lodging varieties, the lodging-resistant varieties had significantly higher mRNA abundance, protein level and enzyme activity levels. The results of this experiment showed that the PAL, 4CL and CAD activities of wheat treated with nano silicon fertilizer were higher than those of CK in each stage, and the relative expression levels of *TaPAL*, *Ta4CL* and *TaCAD* genes were also higher than those of CK. Among them, the three enzyme activities of T2 were the highest and the relative expression levels of the three genes were also the highest, indicating that the application of nano silicon fertilizer was beneficial to enhance the lodging resistance of wheat and the application amount of nano silicon fertilizer in T2 was the best.

Due to the strong adsorption capacity of nano silicon particles, the mixed application with conventional compound fertilizer can make the conventional compound fertilizer slowly release due to the adsorption of nano silicon, effectively prolong the fertilizer effect time and promote the long-term healthy growth of plants [23]. Silicon is beneficial to the growth and development of plants. It has been found that silicon fertilizer can increase the biomass and yield of plants, improve the photosynthesis of plants, improve the utilization rate of P, K, Ca and other nutrients, improve the stress resistance of plants, and alleviate the toxic effects of heavy metals such as Cd, As and Pb [53]. In this study, the effect of nano silicon fertilizer on the lodging resistance of wheat was mainly explored. The two-year experiment showed that the stem breaking-resistance strength, lodging-resistance index and lignin accumulation of wheat treated with nano silicon fertilizer were better than those of CK, and the activities of PAL, 4CL and CAD and the relative expression of related genes were also higher than those of CK at anthesis stage, filling stage and maturity stage. After the application of nano silicon, the fertilizer efficiency time was prolonged, the nutrient leaching was reduced, and the utilization efficiency of fertilizer was improved. However, the application amount of nano silicon was not the more the better. The results of this experiment showed that the application amount of nano silicon in T2 was the best, and its effect on wheat lodging resistance was the best. If too much silicon is applied, it may lead to soil hardening or affect the absorption of other elements. However, in general, the application of appropriate amount of nano silicon is beneficial to the growth of plants.

In summary, the application of appropriate amount of nano silicon is beneficial to improve the lodging resistance of wheat basal second stem node, and it is also beneficial to improve the utilization efficiency of fertilizer, which is of great significance for efficient and reasonable fertilization in agricultural production and the improvement of wheat quality.

## Data Availability

The datasets generated during and/or analysed during the current study are available from the corresponding author on reasonable request.
